# CAD4TB software updates: different triaging thresholds require caution by users and regulation by authorities

**DOI:** 10.5588/ijtld.22.0437

**Published:** 2023-02-01

**Authors:** J. Fehr, R. Gunda, M. J. Siedner, W. Hanekom, T. Ndung’u, A. Grant, C. Lippert, E. B. Wong

**Affiliations:** 1 Africa Health Research Institute, KwaZulu-Natal, South Africa; 2 Digital Health & Machine Learning, Hasso Plattner Institute for Digital Engineering, Potsdam, Germany; 3 Digital Engineering Faculty, University of Potsdam, Potsdam, Germany; 4 School of Nursing and Public Health, College of Health Sciences, University of KwaZulu-Natal, KwaZulu-Natal, South Africa; 5 Division of Infection and Immunity, University College London, London, UK; 6 School of Clinical Medicine, College of Health Sciences, University of KwaZulu-Natal, KwaZulu-Natal, South Africa; 7 Harvard Medical School, Boston, MA, USA; 8 Division of Infectious Diseases, Massachusetts General Hospital, Boston, MA, USA; 9 HIV Pathogenesis Programme, The Doris Duke Medical Research Institute, University of KwaZulu-Natal, Durban, South Africa; 10 Ragon Institute of MGH, MIT and Harvard University, Cambridge, MA, USA; 11 London School of Hygiene & Tropical Medicine, London, UK; 12 School of Clinical Medicine, College of Health Sciences, University of KwaZulu-Natal, KwaZulu-Natal, South Africa; 13 Hasso Plattner Institute for Digital Health at Mount Sinai, Icahn School of Medicine at Mount Sinai, New York, NY, USA; 14 Division of Infectious Diseases, University of Alabama at Birmingham, AL, USA

Dear Editor,

The recent recommendations by the WHO for systematic screening for TB with digital chest X-ray (CXR) and automated imaging interpretation^1^ has led to an explosion in the use of computer-assisted diagnostic (CAD) algorithms. We previously found that the performance of CAD4TB (^©^Delft Imaging, Hertogenbosch, The Netherlands) is comparable to a human radiologist during community-based TB screening in rural South Africa.^2^ CAD4TB quantifies lung field abnormalities suggestive of active TB, assigning a score between 0 and 100. Using CAD4TB requires screening programmes to select a triaging threshold above which participants receive sputum testing. Triaging thresholds are not universal and require adjustment based on demographic characteristics, laboratory capacities, budget, healthcare settings and programmatic goals.^2^–^7^ CAD4TB is updated annually, and the 7^th^ version has been released recently. Screening programmes might be eager to use new versions because studies report improved performance,^8^,^9^ but no recommendations on adopting software updates currently exist. Here, we have evaluated the triaging performance characteristics and optimal thresholds of the latest version of CAD4TB (v7) compared to the two most recent versions (v5 and v6).

During the first year of a community-based multi-morbidity study in a rural district in KwaZulu-Natal, South Africa, 9,912 local residents above 15 years of age received free TB screening at a mobile camp (as described previously).^2^,^10^ Briefly, TB screening included digital posterior-anterior CXR imaging and assessment of symptoms. Following WHO guidelines for TB prevalence surveys,^11^ participants were triaged for sputum collection for any TB-related symptom (fever, weight loss, cough or night sweats) or for any CXR lung field abnormality. CXRs were analysed using CAD4TB v5 and scored between 0 and 100 to indicate the likelihood of TB-related lung field abnormality. As described previously,^2^ those with CAD4TB v5 *>*25 (a triaging threshold with a sensitivity of 85% for lung field abnormality)^2^ were triaged for sputum examination using Xpert® MTB/RIF Ultra (Cepheid, Sunnyvale, CA, USA) and MGIT^™^ (BD, Franklin Lakes, NJ, USA) liquid culture, and defined as microbiologically confirmed TB if either test was positive. Among the 9,912 participants who underwent CXR, 5,594 (56.4%) were referred for sputum testing, 4,976 (89.0%) of whom were able to produce sputum. A total of 99 (1.0%) participants had microbiologically positive sputum. A senior radiologist (blinded to CAD4TB scores and patient information) interpreted CXRs as having normal or abnormal lung fields. CXRs were retrospectively analysed using CAD4TB v6 and v7. The distribution of CAD4TB scores (v5–v7) and percentage of participants required to test were compared among all CXRs (*n* = 9,912). Performance characteristics and triage threshold that most closely matched the radiologist’s performance were compared (v5–v7) among individuals with sputum test results (*n* = 4,976). Participants provided written informed consent to participate in the study. Ethics approval was obtained from the University of KwaZulu-Natal Biomedical Research Ethics Committee (BE560/17), KwaZulu-Natal, South Africa; the London School of Hygiene & Tropical Medicine Ethics Committee (14722), London, UK; and the Mass General Brigham Institutional Review Board, Boston, MA, USA (2018P001802).

The overall performance between CAD4TB v5, v6 and v7 (area under the curve [AUC] v5: 0.78, 95% CI 0.73–0.83; v6: 0.79, 95% CI 0.73–0.84; v7: 0.80, 95% CI 0.75–0.85; *P >* 0.1; Figure Panel A) was similar, but the distribution of scores across the 100-point scale varied greatly across the three versions (median scores were v5: 28, interquartile range [IQR] 22–41; v6: 35, IQR 16–46; and v7: 11, IQR 5.2–27; *P <* 0.001; Figure Panel B). Between the three versions, each numerical threshold had strikingly different performance. For example, triaging with a CAD4TB threshold of 40 would result in a range of screening sensitivities (v5: 79.8%, v6: 88.9%, v7: 66.7%) and specificities (v5: 57.4%, v6: 33.3%, v7: 84.6%). As no threshold from any version met the WHO target product profile of ≥90% sensitivity and ≥70% specificity,^12^ we identified one threshold for each CAD4TB version that most closely matched the radiologist sensitivity at 80.8% (95% CI 71.7–88.0). The matching thresholds were v5: 40 (79.8%, 95% CI 70.5–87.2); v6: 47 (82.8%, 95% CI 73.9–89.7); and v7: 20 (79.8%, 95% CI 70.5–87.2). At these thresholds, the three CAD4TB versions had lower specificity than the radiologist (radiologist: 66.9%, 95% CI 65.6–68.2; v5: 40, 57.4%, 95% CI 56.0–58.8; v6: 47, 62.6%, 95% CI 61.2–64.0; v7: 20, 56.6%, 95% CI 55.2–58.0), leading to a higher percentage of participants who would require microbiological sputum testing relative to all participants (*n*=9,912) (radiologist: 20.2%; v5: 40, 27.0%; v6: 47, 23.7%; v7: 20, 33.5%; Figure Panel C and Supplementary Data S1). Substantial variations were also observed in the number of cases of microbiologically positive sputum that would be ‘missed’ using potential triaging thresholds for the different CAD4TB versions (Figure Panel D). For example, triaging with CAD4TB threshold 40, would result in sputum testing for 27.0% (v5), 45.9% (v6) and 10.3% (v7) of participants. At the same threshold, the percentage of microbiologically confirmed TB cases missed would be 20.2% (v5), 11.1% (v6) and 33.3% (v7). To note, despite previous reports that showed improved performance with newer versions,8,9 in these real-world data v7 did not outperform v6, as measured by AUC and specificity matched at the radiologist sensitivity. Despite similar AUC, v7 performed at higher specificity but lower sensitivity at each triaging threshold compared to v5 and v6 (Supplementary Table S1).

**Figure:**
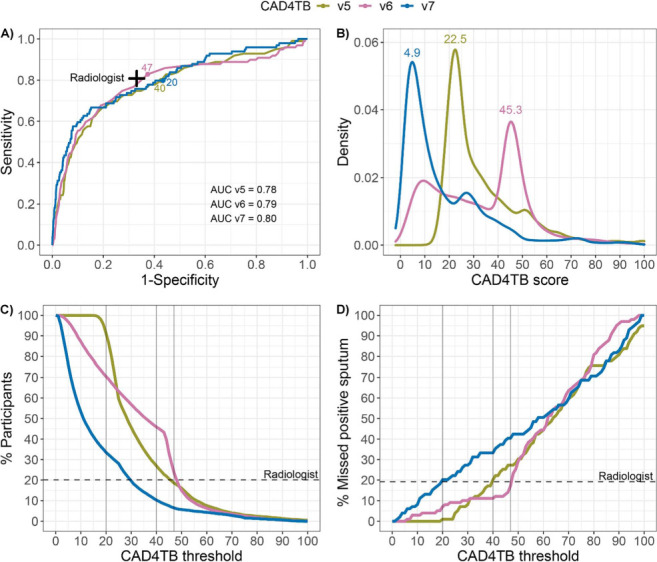
Performance of CAD4TB v5, v6 and v7 to identify microbiologically confirmed TB. TB was defined if sputum was found to be positive on either Xpert Ultra or microbiological culture. A) For individuals with sputum results (n = 4,976), performance is shown in terms of sensitivity and specificity and AUC. Annotations show thresholds that closest matched the radiologist’s sensitivity; B) distributions and most frequent CAD4TB scores of all three versions obtained for all chest X-rays (n = 9,912); C) percentage of participants triaged for sputum testing among all participants at each CAD4TB threshold (n= 9,912); D) percentage of missed positive sputum among TB-positive individuals (n = 99) at each CAD4TB threshold. The performance of the senior radiologist is marked with a cross (A) and dashed lines (C, D). CAD4TB thresholds that matched the radiologist’s performance (v5: 40, v6: 47, v7: 20) are marked with numbers (A) and grey vertical lines (C, D). AUC = area under the receiver-operating curve.

The change in scales and resulting wide variations in triaging thresholds between different CAD4TB versions poses a risk to end-users in TB screening programmes who may unintentionally introduce systematic screening errors by adopting software updates without adjusting the selected triaging thresholds. Using incorrect triaging thresholds may have severe consequences and result in missing people with TB (triage threshold inadvertently too high) or utilising microbiological testing excessively (triaging threshold inadvertently too low). To accommodate intra-version variation, screening programmes need to select new triaging thresholds for each new software update. Previous work^2^,^13^,^14^ and the developer^15^ suggest that it is necessary to conduct pilot studies to finding triage thresholds that optimally serve the goals of each screening exercise. It is now unclear whether each software update requires new piloting for re-adjustment or whether this can be achieved through retrospective analysis of the newest version’s performance against population specific CXR collections. It is unknown whether our findings of significant variation between CAD4TB versions is applicable to other image interpretation algorithms used for TB screening – this information needs to be established urgently.^15^ For anyone designing TB screening programmes, decisions about programmatic adjustments to new versions are especially difficult because the underlying algorithmic or data changes between software versions are not communicated by manufacturers. Changes to the underlying reference standard for algorithm training may require readjustment of triaging thresholds, whereas small changes for faster radiograph interpretation, might not. However, information about the changes between versions is not transparently shared with the community because it has been considered proprietary by developers.^15^

Based on the results presented here, we call for regulation to require CAD-developing companies to communicate changes between software versions and give guidance for medical or public health end-users to effectively adopt software version updates in TB screening programmes. Continued vigilance and performance auditing of successive CAD software versions should be an integral requirement for authorisation by the WHO and regulatory agencies. These findings also contribute to ongoing scientific debates on how to successfully adopt artificial intelligence-based tools for healthcare.
